# Iodide‐Mediated Rapid and Sensitive Surface Etching of Gold Nanostars for Biosensing

**DOI:** 10.1002/anie.202017317

**Published:** 2021-03-24

**Authors:** Yunlei Xianyu, Yiyang Lin, Qu Chen, Alexis Belessiotis‐Richards, Molly M. Stevens, Michael R. Thomas

**Affiliations:** ^1^ College of Biosystems Engineering and Food Science Zhejiang University Hangzhou Zhejiang 310058 China; ^2^ Fuli Institute of Food Science Zhejiang University Hangzhou Zhejiang 310058 China; ^3^ Ningbo Research Institute Zhejiang University Ningbo Zhejiang 315100 China; ^4^ State Key Laboratory of Chemical Resource Engineering Beijing Laboratory of Biomedical Materials Beijing University of Chemical Technology Beijing 100029 China; ^5^ Department of Materials Department of Bioengineering and Institute of Biomedical Engineering Imperial College London London SW7 2AZ UK; ^6^ London Centre for Nanotechnology University College London London WC1H 0AH UK

**Keywords:** gold nanostars, horseradish peroxidase, iodide, plasmonic immunoassay, surface etching

## Abstract

Iodide‐mediated surface etching can tailor the surface plasmon resonance of gold nanostars through etching of the high‐energy facets of the nanoparticle protrusions in a rapid and sensitive way. By exploring the underlying mechanisms of this etching and the key parameters influencing it (such as iodide, oxygen, pH, and temperature), we show its potential in a sensitive biosensing system. Horseradish peroxidase‐catalyzed oxidation of iodide enables control of the etching of gold nanostars to spherical gold nanoparticles, where the resulting spectral shift in the surface plasmon resonance yields a distinct color change of the solution. We further develop this enzyme‐modulated surface etching of gold nanostars into a versatile platform for plasmonic immunoassays, where a high sensitivity is possible by signal amplification via magnetic beads and click chemistry.

## Graphical abstract

We outline a rapid and sensitive nanoparticle etching‐based strategy for biosensing. Iodide modulates the plasmonic features of gold nanostars by etching them into spherical nanoparticles, being a versatile biosensing platform for enzyme detection and plasmonic immunoassays.

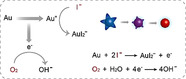

Plasmonic nanomaterials such as gold nanoparticles represent a useful class of nanomaterials that for their size, exhibit exceptionally high extinction coefficients due to their characteristic surface plasmon resonance.^[1]^ These optical properties can be influenced by their size, shape and morphology, providing routes to develop nanosensors with convenient and straightforward absorbance‐based readouts.^[2]^ Three strategies have been developed to modulate the plasmonic signals of nanoparticles and design plasmonic nanosensors: isotropic/anisotropic structural growth, aggregation/disaggregation of nanoparticles, and surface etching.^[3]^ Biosensors built on nanoparticle growth enable a high sensitivity by controlling the nucleation/growth process of nanoparticles. However, the instability issue still remains since the nucleation/growth can be perturbed by the subtle changes of ambient conditions. The aggregation/disaggregation of nanoparticles, especially gold nanoparticles, are approaches widely used for biosensing applications.^[4]^ However, the nonspecific aggregation of nanoparticles as a result of their high specific surface energy may impair the accuracy of the assays.^[5]^ Surface etching holds potential as a prominent way to develop plasmonic nanosensors by modulating the nanoparticle size and morphology, thus achieving controlled plasmonic signals for biosensing applications.^[6]^ Unlike Au growth reaction, the dissolution of Au^0^ by oxidation into Au^I^ is energetically less favored since the oxidation potential of Au^0^ to Au^I^ is as high as 1.425 V.^[7]^ In this sense, biosensor design using surface etching of gold nanoparticles holds promise as a more robust strategy than the other two methods since the plasmonic properties will be less affected by environmental conditions.

In this work, we have reported the iodide‐induced rapid and sensitive surface etching of gold nanostars and developed it into a versatile biosensing platform for enzyme‐catalyzed detections. Gold nanostars exhibit polarization‐dependent scattering and absorption with multiple spectral peaks that are particularly susceptible to variation in the eccentricity of the protruding tips.^[8]^ Herein, we show that reshaping of these tips by iodide can rapidly and sensitively yield a blue‐shift of the surface plasmon resonance (SPR) spectrum in an enzyme‐dependent manner suitable for bioassays.^[9]^

Gold nanostars prepared with an average size of 70 nm via a seed‐mediated method in HEPES buffer exhibited two distinct SPR bands including an intense longitudinal and a weaker transverse plasmon band at 780 and 550 nm, respectively (Supporting Information, Figure S1).^[10]^ The tips of anisotropic plasmonic nanomaterials are known to be highly reactive due to their high surface energy.^[11]^ We characterized the influence of different concentrations of iodide on the nanostar morphology by transmission electron microscope (TEM, Figure 1 a–c). In the absence of iodide, we observed gold nanostars with multiple high eccentricity branches. When exposed to a low concentration of iodide (1 μM, Figure 1 b), gold nanostars became less branched nanostructures with rounded tips. In contrast, when incubated with a high concentration of iodide (10 μM, Figure 1 c), gold nanostars were etched to form spherical nanostructures. Dynamic light scattering revealed a difference in the diffusion mode of the gold nanostars before and after etching (Supporting Information, Figure S2). The asymmetric gold nanostars displayed two hydrodynamic size distributions corresponding to a translational diffusion mode and a rotational diffusion mode, respectively, while the spherical nanostructures formed after etching only showed one hydrodynamic radius representing a translational diffusion mode.^[12]^

**Figure 1:**
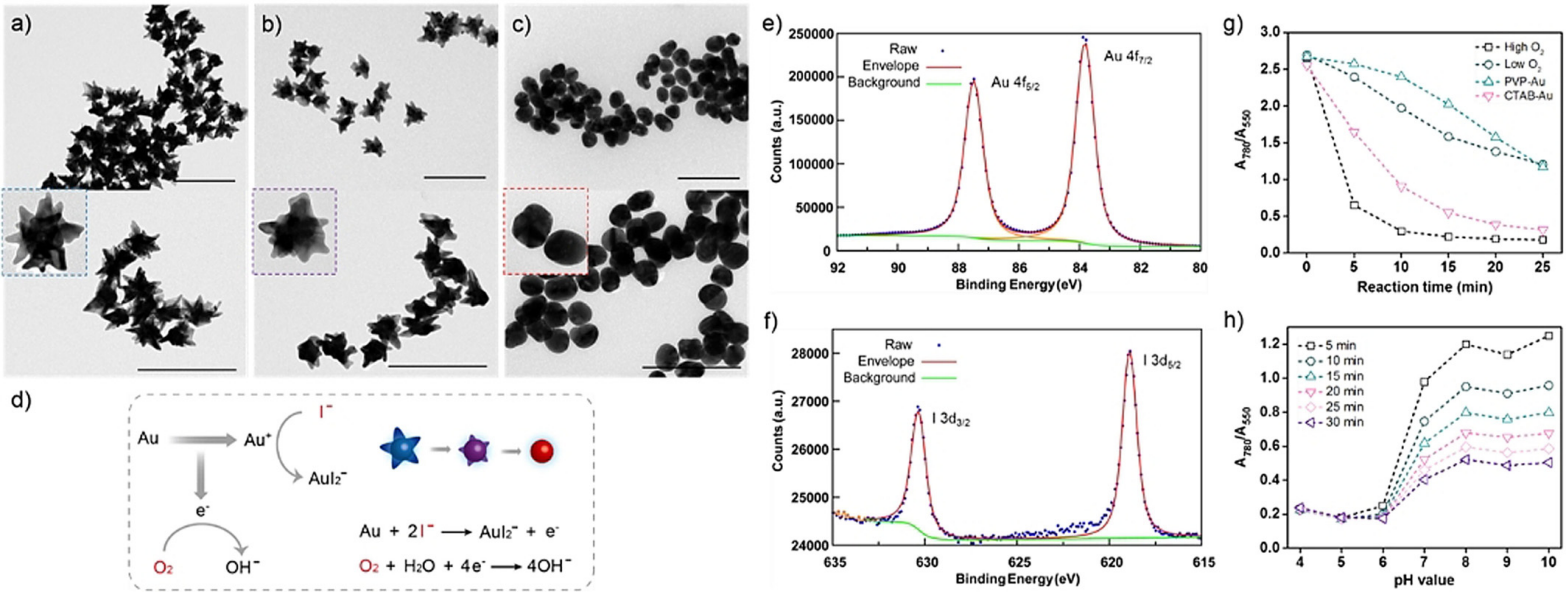
a)–c) TEM images showing iodide‐mediated surface etching of gold nanostars: a) Native gold nanostars. b) Gold nanostars etched by 1 μM iodide. c) Gold nanostars etched by 10 μM iodide. Scale bar: 200 nm. d) Illustration of the proposed mechanism and morphological change during the surface etching of gold nanostars. e) High‐resolution XPS spectra of Au 4f peaks before etching. f) High‐resolution XPS spectra of I 3d peaks after etching. g) Time‐dependent A_780_/A_550_ value of gold nanostars incubated with 2 μM iodide to indicate the effect of oxygen and capping ligands on the nanostar etching kinetics. h) Time‐dependent A_780_/A_550_ value of gold nanostars incubated with 2 μM iodide at pH values ranging from 4 to 10.

The surface etching of gold nanostars is understood to proceed via an electrochemical reaction at the interface where I^−^, O_2_, the pH, and the temperature are crucial (Figure 1 d).^[13]^ There are two essential components in the etching process: a complexing ligand (I^−^) and an oxidant (O_2_).^[14]^ Thermodynamically, surface etching of gold without a strong ligand to form a stable Au^I^ complex is impossible since Au is highly stable. The complexation of gold and iodide can occur at the interface due to the high affinity of iodide to Au.^[15]^ According to Le Chatelier's principle, the binding of ligands to oxidized Au ions or the formation of a stable Au complex will increase the yield of oxidation reactions. Since Au atoms are “soft” Lewis acids, their complexation with iodide, referred to as a typical “soft” Lewis donor, will be energetically favored. Indeed, the affinity of iodide to the surface of gold nanostars was confirmed by the successive decrease of ζ potential from −42 mV to −50 mV upon the incubation with iodide (Supporting Information, Figure S3). We also confirmed the adsorption of iodide by X‐ray photoelectron spectroscopy (XPS) which showed only the Au 4f peaks prior to etching but both the I 3d peaks and Au 4f peaks after etching (Figure 1 e,f; Supporting Information, Figure S4). The strong affinity of iodide to gold nanostars led to the surface etching and thereby a plasmonic peak shift from 780 nm to 550 nm (Supporting Information, Figure S5). We used the A_780_/A_550_ value to indicate the degree of surface etching, and a lower A_780_/A_550_ value suggested a higher degree of etching.^[16]^ According to the proposed mechanism, the dissolved oxygen acted as the oxidant in the etching process that enabled Au oxidation at the nanoparticle‐solution interface. In a separate experiment, we conducted a reaction under similar conditions except that we flushed the reaction solution with argon to remove oxygen. As shown in Figure 1 g and the Supporting Information, Figure S5, we found that the etching process was impaired in an oxygen‐depleted solution, revealing the key role of oxygen. The benefit of using a nanostar synthetic method employing only HEPES is highlighted in the distinct impact of other more common capping ligands on the nanostar etching kinetics. Compared with polyvinylpyrrolidone (PVP)‐capped or cetyltrimethylammonium bromide (CTAB)‐capped nanostars, particularly rapid etching of the unmodified gold nanostars was observed (Figure 1 g). HEPES acted as both the buffer and the capping ligand to stabilize gold nanostars. The advantage of using merely HEPES was that it enabled rapid etching which could contribute to a rapid response for the biosensor development. Compared with other capping ligands, HEPES was a much smaller molecule that led to a higher accessibility of iodide towards the surface of gold nanostars. Consequently, the high accessibility resulted in rapid etching kinetics. Compared with previous work such as CTAB‐mediated nanoparticle reshaping that required several hours, the iodide‐mediated surface etching could be completed within 20 minutes.^[17]^ Furthermore, the concentration of hydroxide ion or the pH could affect the surface etching according to the etching mechanism. At a low pH value, hydroxide ions generated from the electrochemical reaction were consumed immediately by neutralization and as a result the position of equilibrium moved to the right. As expected, we found that the surface etching was greater at a pH value below 6 (Figure 1 h; Supporting Information, Figure S6). Since the electrochemical reaction was mass transport limited, we could achieve a higher overall etching rate by increasing the diffusion rate of the ligand or oxidant towards the surface, which was obtained by increasing the reaction temperature (Supporting Information, Figure S7).

We performed scanning transmission electron microscopy (STEM) and energy‐dispersive X‐ray spectroscopy (EDS) measurements to map the spatial elemental distribution of Au, S, and I before and after etching by iodide (Figure 2; Supporting Information, Figure S8). High‐angle annular dark‐field STEM (HAADF‐STEM) images showed that native gold nanostars displayed distinct nanostructures including the gold core and multiple protruding tips (Figure 2 a,b). In contrast, when we incubated the gold nanostars with 10 μM iodide their surface was etched to form spherical nanostructures (Figure 2 c,d). EDS mapping revealed that both the initial gold nanostars and the spherical nanostructures were enriched with Au and S that originated from HEPES due to the formation of Au−S bond. However, the spherical nanostructures produced from etching appeared to present more iodide compared with the nanostars, further suggesting that iodide was likely binding to the surface during etching of the tips of nanostars.

**Figure 2:**
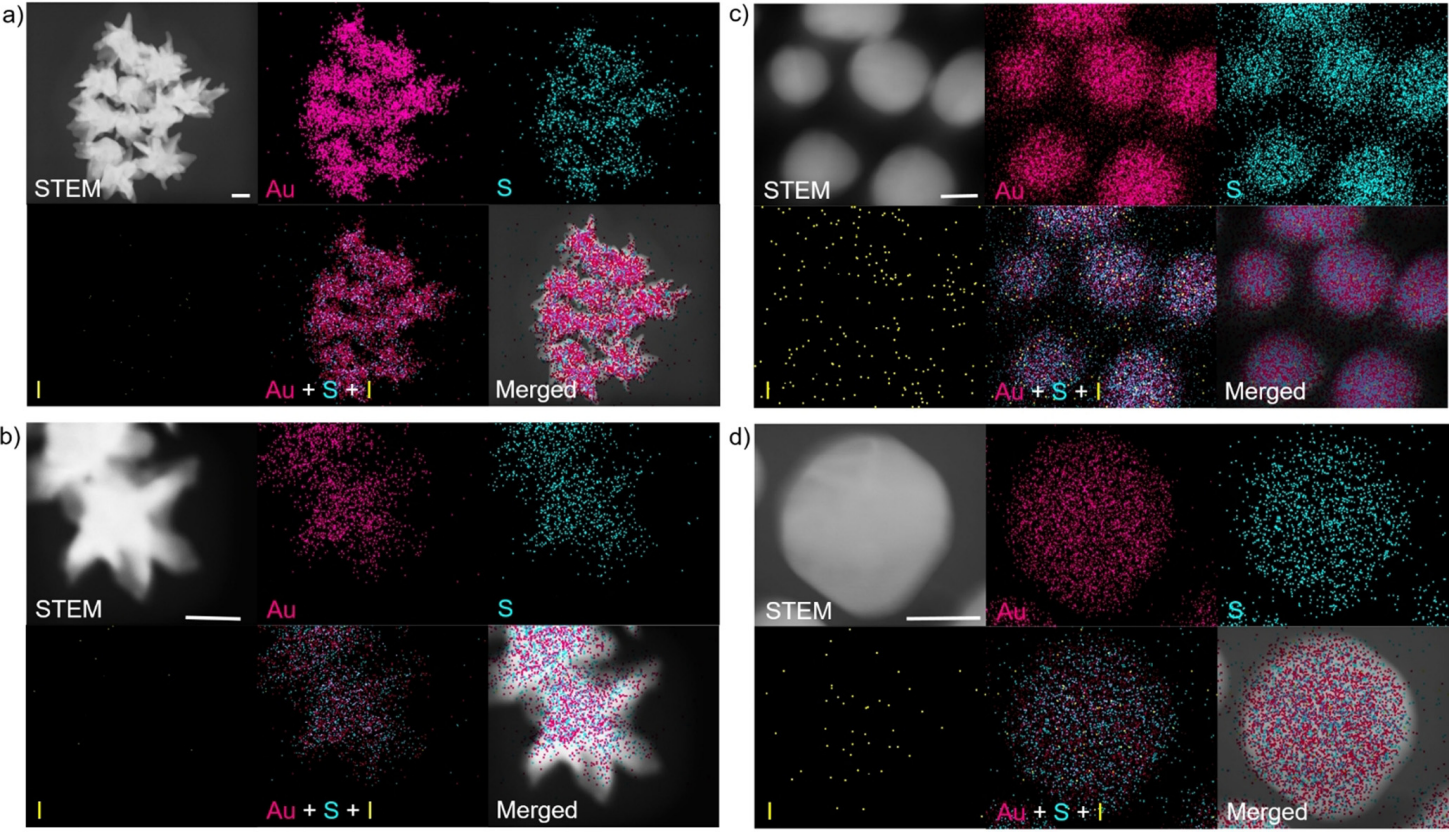
HAADF‐STEM images and EDS mapping of the spatial distribution of Au, S, and I before and after the iodide‐mediated etching of gold nanostars. a),b) HAADF‐STEM images and EDS mapping of native gold nanostars and single gold nanostar before etching. c),d) HAADF‐STEM images and EDS mapping of spherical nanoparticles and single gold nanoparticle after etching by 10 μM iodide. Scale bar: 25 nm.

We incubated gold nanostars with different concentrations of iodide for a fixed time interval (20 min) while monitoring their SPR response. The spectra obtained showed that as the concentration of iodide was increased, the longitudinal plasmon band at 780 nm underwent a blue shift and the transverse plasmon band at 550 nm increased in absorbance (Figure 3 a,b). The blue‐shift of the spectrum could be attributed to a higher degree of etching by a higher concentration of iodide. Following exposure to 1.1 μM iodide, gold nanostars were etched to present a single plasmon band at around 550 nm. Owing to the blue‐shift of the plasmon spectrum in the visible region (400–800 nm), the color of the solution changed from blue to purple and finally red. Such a color change could be readily visualized by the naked eye, allowing for an instrumentation‐free readout for the qualitative detection of iodide. The surface etching of gold nanostars was particularly iodide‐sensitive and even a 20 nM difference in the concentration could be discriminated (Figure 3 c; Supporting Information, Figure S9). The high sensitivity of the surface etching was due to the unique structure of the gold nanostars and their capping surfactant‐ and polymer‐free surfaces. Etching of the protruding tips of nanostars led to a significant spectral shift from 780 nm to 550 nm while etching of gold nanospheres did not result in a peak shift (Supporting Information, Figure S10). When unmodified gold nanostars were blocked by PVP, the surface etching was found to be impaired potentially due to the reduced accessibility of iodide to the surface of gold nanostars (Supporting Information, Figure S11).

**Figure 3:**
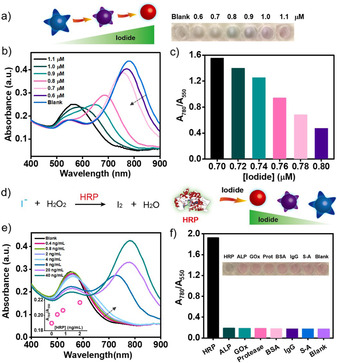
Sensitivity and specificity of iodide‐mediated surface etching for HRP sensing. a) Illustration and photograph of concentration‐dependent surface etching of gold nanostars by iodide. b) UV/Vis spectra of gold nanostars etched by iodide ranging from 0.6 μM to 1.1 μM. c) A_780_/A_550_ value of gold nanostars etched by iodide ranging from 0.7 μM to 0.8 μM. d) Illustration of HRP‐catalyzed reaction for iodide consumption that prevents the surface etching of gold nanostars. e) UV/Vis spectra of gold nanostars after the iodide consumption catalyzed by HRP ranging from 0.4 ng mL^−1^ to 40 ng mL^−1^. Inset: *A*_780_/*A*_550_ value corresponding to the HRP concentration (0, 0.4, 0.8, 2, 4 ng mL^−1^). f) Photograph and A_780_/A_550_ value of gold nanostars after the iodide consumption by the catalysis of HRP (40 ng mL^−1^) and other proteins (800 ng mL^−1^) including ALP, GOx, protease (Prot), BSA, IgG, and streptavidin (S‐A).

We demonstrated that iodide can selectively etch gold nanostars by incubating them with other anions including CO_3_^2−^, NO_3_^−^, SO_4_^2−^, HPO_4_^2−^, H_2_PO_4_^−^, C_6_H_7_O_6_^−^, ClO_2_^−^, IO_4_^−^, Cl^−^, and Br^−^ at a concentration of 20 μM (Supporting Information, Figure S12). The plasmon bands of gold nanostars incubated with these anions remained unchanged compared with the native gold nanostars. In contrast, the spectra of gold nanostars incubated with iodide (2 μM) showed a dramatic blue‐shift from 780 nm to 550 nm. In the case of other halides, we observed no surface etching of gold nanostars with Br^−^ and Cl^−^ in agreement with the previous findings and attributed to their small solvation shell.^[18]^ This is because halide ions are known to have different affinities to gold surfaces with binding energies that scale with polarizability (I^−^ > Br^−^ > Cl^−^) and crystal facet ((111) > (110) > (100)).^[19]^

We coupled the iodide‐specific surface etching of gold nanostars with an enzyme‐catalyzed iodide oxidation reaction to generate an enzyme biosensor, where the biological signal could be transduced into optical responses. Horseradish peroxidase (HRP), as one of the most widely used enzymes in bioassays, is known to catalyze the reaction of iodide oxidation into iodine in the presence of hydrogen peroxide (H_2_O_2_) under acidic conditions (Figure 3 d). Unlike iodide, the oxidation product iodine was incapable of etching gold nanostars at concentrations between 1.0 μM and 1.5 μM (Supporting Information, Figure S13). Neither H_2_O_2_ nor hydrochloric acid (up to 100 μM) was capable of etching gold nanostars (Supporting Information, Figure S14). In contrast, iodide could etch gold nanostars resulting in a blue‐shift of the plasmon spectrum under acidic conditions (Supporting Information, Figure S15). By combining the HRP‐catalyzed iodide oxidation and iodide‐mediated gold etching reaction, we were able to detect HRP by monitoring shifts in the plasmonic spectrum of the nanostars. In the absence of HRP, iodide‐induced etching of nanostars resulted in spherical nanostructures and a red color of the solution. In the presence of increasing concentrations of HRP, more iodide was consumed which led to the alleviation of the surface etching and a blue color of the solution. Since gold nanostars feature characteristic plasmonic spectral responses in both position and absorbance as a function of analyte concentration, we directly used the lowest concentration assayed whose spectrum could be differentiated from that of the blank samples to evaluate the sensitivity of the assays. This was identified as the first response in A_780_/A_550_ value greater than the blank response plus three times the standard deviation. This etching‐based method enabled the detection of 0.4 ng mL^−1^ HRP (Figure 3 e). The specificity of the HRP sensor was tested for several proteins and enzymes such as alkaline phosphatase (ALP), glucose oxidase (GOx), protease (Prot), bovine serum albumin (BSA), immunoglobulin G (IgG) and streptavidin (S‐A) none of which were found to yield a similar response (Figure 3 f; Supporting Information, Figure S16).

We further developed this gold etching‐based HRP sensor into an enzyme‐linked immunosorbent assay (ELISA). To enhance the sensitivity of this assay, we employed magnetic beads (MBs) co‐functionalized with antibodies and HRP via click chemistry to increase the number of HRP enzymes bound per target analyte (Figure 4). We used a 2‐step methodology to prepare the antibody presenting HRP‐coated MBs (Ab‐HRP‐MBs) (Figure 4 a). Initially, we conjugated HRP to the MBs (HRP‐MBs) via the reaction between N‐hydroxysuccinimide (NHS)‐activated carboxyl groups on MBs and the amine groups on HRP. HRP‐MBs were functionalized with 1,2,4,5‐tetrazine (Tz) by reacting with NHS‐Tz, which was further conjugated with *trans*‐cyclooctene (TCO) functionalized antibodies. The bioorthogonal copper‐free cycloaddition reaction between Tz and TCO has been widely applied for biological functionalization and has been shown to impose minimal perturbation of biological activities.^[20]^ We confirmed the conjugation of Tz groups onto HRP‐MBs by the interaction between the conjugates and TCO‐FAM, showing that the HRP‐MBs were fluorescently labeled which indicated the successful preparation of Tz‐HRP‐MBs and the occurrence of click reaction between TCO and Tz (Supporting Information, Figure S17). The specific number of HRP loaded on one MB was calculated to be ca. 26 530 which was close to previous studies (Supporting Information, Figure S18).^[21]^ The Ab‐HRP‐MBs not only acted as the detection probe that could specifically recognize the target molecules, but also acted as the signal amplifier that could catalyze the consumption of iodide for the etching‐based readout due to the large loading amount of HRP molecules per MB.

**Figure 4:**
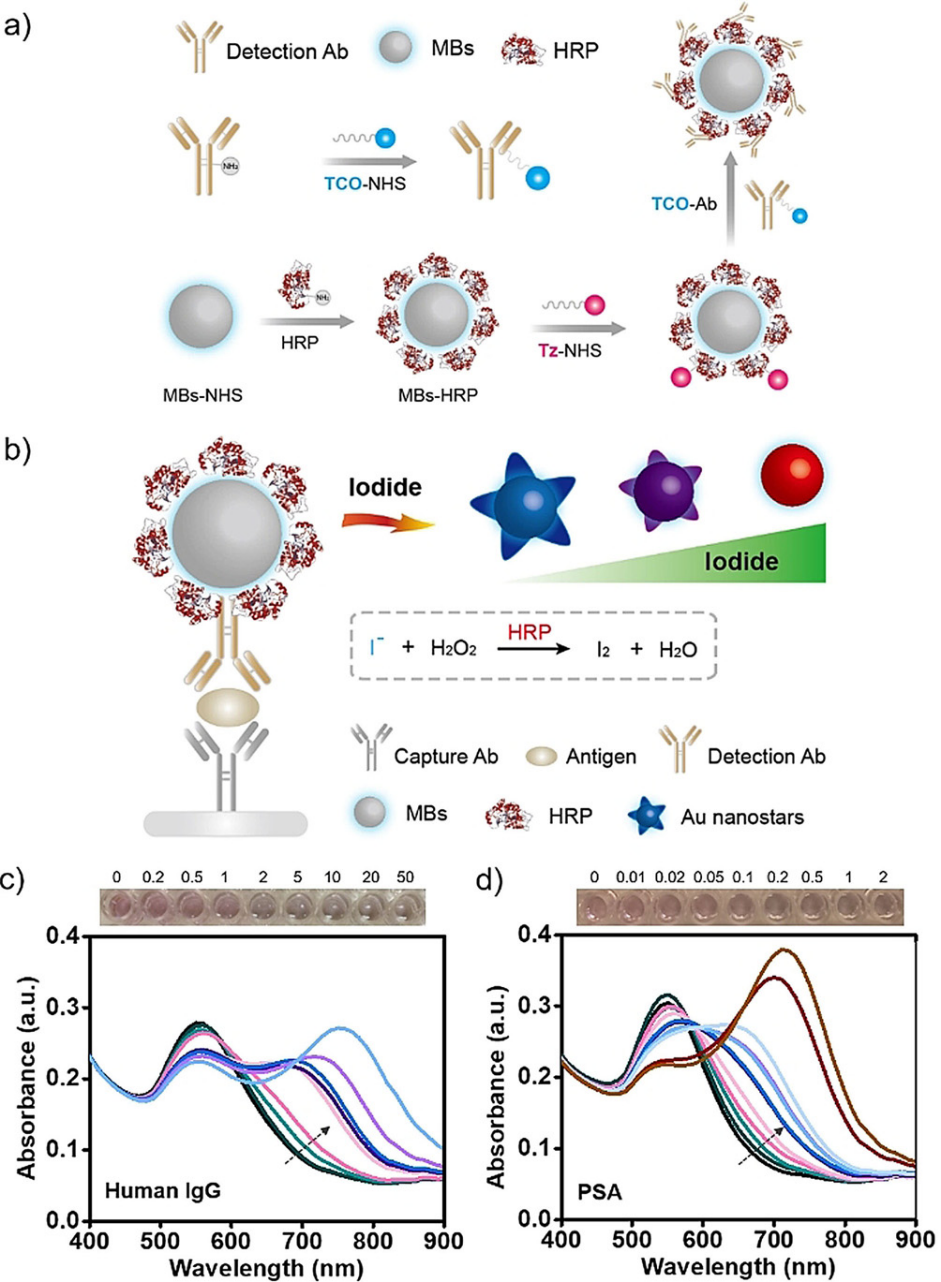
Iodide‐mediated surface etching of gold nanostars for plasmonic immunoassays. a) Illustration of the 2‐step procedure for preparation of Ab‐HRP‐MBs using TCO‐Tz click chemistry. b) Illustration of the surface etching‐based plasmonic immunoassay that combines HRP‐catalyzed oxidation of iodide and TCO‐Tz click chemistry. c),d) Photographs and UV/Vis spectra of gold nanostars after c) human IgG and d) PSA detections using the etching‐based plasmonic immunoassay. The concentrations of human IgG are 0.2, 0.5, 1, 2, 5, 10, 20, 50 ng mL^−1^ (from the black line to the light blue line). The concentrations of PSA are 0.01, 0.02, 0.05, 0.1, 0.2, 0.5, 1, 2, 5, 10, 20 ng mL^−1^ (from the black line to the brown line).

We developed HRP‐modulated surface etching of gold nanostars into a versatile platform for plasmonic immunoassays (Figure 4 b). As a proof of concept, we used it to detect a model protein (human IgG). In the sandwich immunoassay, goat anti‐human IgG acted as the capture antibody and rabbit anti‐human IgG acted as the detection antibody. The detection antibody and HRP were co‐functionalized onto the MBs for the binding of human IgG and the catalysis of the iodide‐consumed reaction for the detection of human IgG (Figure 4 c). In the absence of human IgG and binding of Ab‐HRP‐MBs, the plasmon spectra of gold nanostars exhibited a characteristic band at 550 nm and the solution showed red coloration. As the concentration of human IgG increased, more Ab‐HRP‐MBs bound to the plate to catalyze the consumption of iodide to prevent the surface etching of gold nanostars enabling IgG detection as low as 0.2 ng mL^−1^. The plasmon spectra gradually presented both a transverse plasmon band at 550 nm and a longitudinal plasmon band towards 780 nm yielding purple or blue solutions. Beyond human IgG detection, we employed this etching‐based method to detect an exemplar clinically relevant protein biomarker, prostate‐specific antigen (PSA). PSA is a biomarker of value for early detection of biochemical recurrence following radical prostatectomy. PSA levels that rise in excess of 0.2 ng mL^−1^ can be indicative of recurrence.^[22]^ Consistent with IgG, the plasmon spectra were blue‐shifted with increasing concentrations of PSA (Figure 4 d; Supporting Information, Scheme S1). Indeed, concentrations as low as 10 pg mL^−1^ could be detected via nanostar etching, although the sensitivity of this etching‐based approach could be potentially enhanced if a longer etching time is implemented. A comparison between this etching‐based assay and other detection methods is listed in the Supporting Information, Table S1.

In conclusion, we report a rapid and sensitive surface etching‐based approach that employs iodide for tailoring the morphology and plasmonic properties of unmodified gold nanostars for the biosensor development. Gold nanostars were etched to form spherical nanostructures with a concomitant modulation of the plasmonic spectrum and resulting color of the nanoparticle solution. The surface etching‐based approach showed a high specificity with a straightforward readout with high sensitivity. We envisage that this etching approach could be useful in a myriad of applications ranging from nanoparticle synthesis to point‐of‐care biomedical diagnostics.

## Conflict of interest

The authors declare no conflict of interest.

## Supporting information

As a service to our authors and readers, this journal provides supporting information supplied by the authors. Such materials are peer reviewed and may be re‐organized for online delivery, but are not copy‐edited or typeset. Technical support issues arising from supporting information (other than missing files) should be addressed to the authors.

Supplementary
