# Supplementary material for: Iodide‐Mediated Rapid and Sensitive Surface Etching of Gold Nanostars for Biosensing
Source: Angew Chem Int Ed Engl. 2021 Mar 24;60(18):9891–6. doi: 10.1002/anie.202017317 (PMC8251757; doi:10.1002/anie.202017317)
Supplement: Supplementary file 1 — Supplementary [file ANIE-60-9891-s001.pdf]

Angewandte Chemie
Eine Zeitschrift der Gesellschaft Deutscher Chemiker

Supporting Information

Iodide-Mediated Rapid and Sensitive Surface Etching of Gold Nanostars for Biosensing

Yunlei Xianyu, Yiyang Lin, Qu Chen, Alexis Belessiotis-Richards, Molly M. Stevens, and Michael R. Thomas*

anie_202017317_sm_miscellaneous_information.pdf

Content List for Supporting Information

Materials and methods -- S-2

Figures

Figure S1 -- S-9

Figure S2 -- S-10

Figure S3 -- S-11

Figure S4 -- S-12

Figure S5 -- S-13

Figure S6 -- S-14

Figure S7 -- S-15

Figure S8 -- S-16

Figure S9 -- S-17

Figure S10 -- S-18

Figure S11 -- S-19

Figure S12 -- S-20

Figure S13 -- S-21

Figure S14 -- S-22

Figure S15 -- S-23

Figure S16 -- S-24

Figure S17 -- S-25

Figure S18 -- S-26

Scheme S1 -- S-27

Table S1 --- S-28

References -- S-29

Material and methods

Materials and instrumentation

Chloroauric acid ($HAuCl_4{\cdot}3H_2O$), 4-(2-hydroxyethyl)-piperazine-1-ethane-sulfonic acid sodium salt (HEPES), hydroxylamine hydrochloride, sodium iodide, hydrogen peroxide and horseradish peroxidase were purchased from Sigma-Aldrich. Tetrazine-PEG_5-NHS ester, *trans*-cyclooctene-PEG_4-NHS ester and tetrazine-5-FAM were purchased from Jena Bioscience GmbH. NHS-activated magnetic beads (NHS-MBs) (1000 nm in size, solid content: 10 mg/mL) and 1-Step Ultra TMB-ELISA substrate solution were purchased from Thermo Fisher Scientific. Human IgG, rabbit anti-human IgG and goat anti-human IgG were purchased from Jackson ImmunoResearch (USA). Anti-prostate specific antigen antibody (ab19554), prostate specific antigen and anti-prostate specific antigen antibody (ab10184) were purchased from Abcam. All the other reagents required for the experiments were of analytical grade and used as received. All solutions were prepared using ultrapure water through a Millipore Milli-Q water purification system with an electric resistance >18.3 MΩ. The UV-Vis absorbance spectra of the solutions of gold nanostars were measured on a SpectraMax M5 plate reader. The average size distribution and zeta potentials of gold nanostars were determined using a Malvern Zetasizer (Nano-ZS, USA). TEM characterizations of gold nanostars were performed on a JEOL 2100Plus or JEOL 2100F TEM at an acceleration voltage of 200 kV.

Synthesis of gold nanostars

Gold nanostars were prepared using a seed-mediated growth method. The seed solution was prepared as follows: $HAuCl_4$ solution (1 mM, 100 mL) was heated under reflux with stirring, followed by the addition of trisodium citrate solution (38.8 mM, 10 mL). The solution was cooled and filtered by a 0.22 μm nitrocellulose membrane for further use. For the synthesis of nanostars, 750 μL of the above citrate-stabilized seed solution was added to 750 μL of 40 mM hydroxylamine hydrochloride, followed by the addition of 18.75 mL of 100 mM HEPES (pH = 8.5) and 38.5 mL of water. The solution was allowed to mix under rigorous stirring (1450 rpm). Then, 22.5 mL of 1 mM chloroauric

acid ($HAuCl_4$) solution was added to the above solution drop wise at room temperature. After that, the solution was allowed to further react for 15 minutes under moderate stirring (700 rpm), followed by the centrifugation (4000 rpm, 30 minutes) for three times at 8 ^{o}C. Finally, the solution was re-dispersed in HEPES and filtered by a 0.22 μm nitrocellulose membrane, and kept at 4 °C for long-term storage.

Surface etching of gold nanostars by iodide

In a typical etching experiment, 10 μL of the prepared gold nanostars were directly incubated with different concentrations of sodium iodide in water or the buffer solution to reach a final volume of 200 μL. The mixture of the solution was allowed to react for 15-20 minutes for the etching of gold nanostars. The surface plasmon resonance (SPR) absorption of gold nanostars was measured by the plate reader. For the investigation of the etching mechanism, chemically inert argon gas was introduced to study the effect of oxygen on the surface etching of gold nanostars. The pH value of the solution was adjusted by 1 M NaOH or HCl to study the effect of pH on the surface etching process. Also, the etching experiment was carried out under different temperatures to study the effect of temperature on the surface etching process.

Surface etching of PVP-capped and CTAB-capped gold nanostars by iodide

PVP-capped gold nanostars were prepared by incubating unmodified gold nanostars with 1 mM PVP (average molecular weight 10000) for one hour and further removing excess PVP by centrifugation for three times. CTAB-capped gold nanostars were prepared by incubating unmodified gold nanostars with 1 mM CTAB. For the etching experiment, 10 μL of the PVP-capped or CTAB-capped gold nanostars were directly incubated with sodium iodide in water to reach a final volume of 200 μL. The final concentration of iodide was 2 μM. The absorbance at 780 nm and 550 nm of gold nanostars was measured by the plate reader every 5 minutes to study the etching kinetics.

Electron microscopy characterisation of gold nanostars

For electron microscopy characterisation, a drop of gold nanostars or the mixture that

contained gold nanostars and iodide was deposited onto the carbon-coated 200 mesh copper grid for 5 minutes. The excess solution was removed using filter paper and the sample was dried in air. TEM was performed on a JEOL 2100Plus or a JEOL 2100F with an acceleration voltage of 200 kV. High angle annular dark field (HAADF) images were acquired in scanning transmission electron microscopy (STEM) mode on a JEOL 2100F operating at 200 kV, equipped with Gatan Orius SC 1000 and high-angle annular dark-field detectors. Elemental compositional mapping of gold nanostars was determined by energy-dispersive X-ray spectroscopy (EDS) in STEM mode.

XPS characterisation of gold nanostars

To prepare the XPS samples, gold nanostars were centrifuged at 5000 rpm for 10 minutes to remove excess HEPES in the initial solution. After incubation with 5 μM of iodide, the gold nanostars were centrifuged again at 5000 rpm for 10 minutes to remove excess iodide and a drop of gold nanostars was placed on a cleaned silicon wafer and air-dried at room temperature. XPS spectra were obtained on a Thermo Fisher K-Alpha$^+$ spectrophotometer which employs a monochromatic Al-Kα X-ray source (energy = 1486.6 eV) and a 180° double focusing hemispherical analyser with a 2D detector. The X-ray source was operated at 6 mA emission current and 12 kV anode bias. Using a 300 μm^2 X-ray spot size, survey spectra were recorded for each sample at pass energies of 200 eV followed by high-resolution measurements for Au 4f, I 3d, and C 1s core levels at pass energies of 20 eV. A flood gun was employed to minimize sample charging during analysis. Curve fitting and data analysis was carried out using the Avantage Software package (v.5.949).

Surface etching of gold nanostars by sodium salts with different anions

Surface etching was studied by mixing gold nanostars with sodium salts with different anions. Briefly, 10 μL of gold nanostars were separately incubated with different sodium salts (Na_2CO_3, $NaNO_3$, Na_2SO_4, Na_2HPO_4, NaH_2PO_4, $NaC_6H_7O_6$, $NaClO_2$, $NaIO_4$, NaCl, NaBr and NaI) to reach a final volume of 200 μL. The concentration of NaI was 2 μM and the concentrations of the other sodium salts was 20 μM. The mixture

of the solution was allowed to react for about 15-20 minutes for the etching of gold nanostars. The surface plasmon resonance (SPR) absorption of gold nanostars was measured using a plate reader (SpectraMax M5, Molecular Devices).

Surface etching of gold nanostars for HRP detection

For HRP detection, hydrogen peroxide (2 mM), sodium iodide (1.1 mM) and different concentrations of HRP were incubated in the buffer solution (pH = 3) to reach a final volume of 100 μL. The mixture was kept to react at 37 °C for 1 hour. 20 μL of the mixture were then used for surface etching of gold nanostars. The SPR absorption of gold nanostars was measured using a plate reader. The specificity of the HRP detection was investigated using 0.2 ng/mL of HRP, alkaline phosphatase, glucose oxidase, protease, bovine serum albumin, immunoglobulin G (IgG) and streptavidin for the reaction between hydrogen peroxide and sodium iodide. After the reaction, the solution mixture was used for surface etching of gold nanostars. The SPR absorption of gold nanostars was measured using a plate reader (SpectraMax M5, Molecular Devices).

Conjugation of tetrazine to the detection antibody

Tetrazine-PEG$_5$-NHS ester was dissolved in DMSO. The antibody (rabbit anti-human IgG or anti-prostate specific antigen antibody) was prepared in 500 μL of PBS (pH = 7.4) at a concentration of 1.0 mg/mL, which was further mixed with 16.5 μL of 20 mM tetrazine-PEG$_5$-NHS ester and incubated at room temperature for 2 hours. The reaction was quenched by adding 1 M Tris-HCl (pH = 8) to reach a final concentration of 50 mM. The product was then purified to obtain tetrazine-antibody by repeatedly using a centrifugal filter device (Amicon Ultra-0.5, Millipore) with a 10K Nominal Molecular Weight Limit (NMWL).

Conjugation of *trans*-cyclooctene to HRP and MBs

HRP was first conjugated to the NHS-MBs through NHS chemistry, followed by the conjugation of *trans*-cyclooctene-PEG$_4$-NHS ester to HRP-MBs. The sequential conjugation process was as follows: 100 μL of NHS-MBs (10 mg/mL) were mixed with

200 μL of HRP (2 mg/mL) in PBS and incubated for 2 hours at room temperature under mild rotation. The HRP-MBs were purified and collected by a magnetic stand, and the supernatant that contained excess HRP was collected for calculating the amount of the loaded HRP per bead. Before the conjugation of *trans*-cyclooctene-PEG_4-NHS ester to HRP-MBs, the HRP-MBs were first quenched with 10 μL of 100 mM glycine for 2 hours to block the unreacted NHS ester. After the magnetic purification, the HRP-MBs were functionalised with *trans*-cyclooctene by adding 2 μL of 20 mM *trans*-cyclooctene-PEG_4-NHS ester for 2 hours. The mixture was incubated for 2 hours at room temperature under mild rotation. The TCO-HRP-MBs were purified and collected on a magnetic stand.

Conjugation of TCO-HRP-MBs with tetrazine-antibody

The conjugation of TCO-HRP-MBs with tetrazine-antibody is based on the Diels-Alder cycloaddition reaction between *trans*-cyclooctenes (TCO) and tetrazines. 1 mL of 1 mg/mL TCO-HRP-MBs were mixed with 10 μL of 1 mg/mL tetrazine-antibody. The mixture was incubated at room temperature for 1 hour under mild rotation. The resulting antibody-conjugated HRP-MBs were purified and collected on a magnetic stand for further use.

Conjugation of TCO-HRP-MBs with tetrazine-FAM

To validate that *trans*-cyclooctenes (TCO) groups have been successfully labelled onto HRP, the HRP-MBs were allowed to incubate with tetrazine-terminated fluorescein (tetrazine-FAM). 100 μL of 1 mg/mL TCO-HRP-MBs were mixed with 10 μL of 1 mg/mL tetrazine-FAM. The mixture was incubated at room temperature for 1 hour under mild rotation. The resulting product was purified and collected on a magnetic stand for fluorescent imaging.

Calculation of loaded HRP molecules on MBs

The amount of loaded HRP molecules per bead can be calculated based on the amount of HRP conjugated on the MBs and the concentration of MBs, where the loaded HRP

can be determined by the initial HRP added in the conjugation process and the excess HRP collected from the supernatant. The enzymatic activity of HRP was measured by using the 1-Step Ultra TMB-ELISA substrate solution. According to a standard curve that was made with a known concentration of HRP, the number of HRP molecules in the supernatant was determined. The amount of HRP loaded on the MBs was calculated to be 2.653×10^{13} HRP/mL, thus the number of HRP molecules per MB was estimated to be 26530 based on the concentration of the MBs (10^{9} particles/mL).

Human IgG immunoassay

Goat anti-human IgG antibody (5 μg/mL) in bicarbonate buffer (100 mM, pH = 9.6) was added into a 96-well polystyrene plate and incubated at 4 °C overnight. The plate was washed and blocked with 5% bovine serum albumin at 37 °C for 1 hour. Different concentrations of human IgG were added and incubated at 37 °C for 30 minutes, followed by washing and addition of antibody-conjugated HRP-MBs. The solution mixture was incubated at 37 °C for 30 minutes, followed by washing the wells three times. After that, hydrogen peroxide and sodium iodide in the buffer solution (pH = 3) were added to reach a final volume of 100 μL, and kept to react at 37 °C for 1 hour. The mixture was then taken out for the etching experiment, and the SPR absorption of gold nanostars was measured using a plate reader (SpectraMax M5, Molecular Devices).

Prostate specific antigen immunoassay

Anti-prostate specific antigen antibody (ab19554, 5 μg/mL) in bicarbonate buffer (100 mM, pH = 9.6) was added into a 96-well polystyrene plate and incubated at 4 °C overnight. The plate was washed and blocked with 5% bovine serum albumin at 37 °C for 1 hour. Different concentrations of prostate specific antigen diluted in PBS were added and incubated at 37 °C for 30 minutes, followed by washing and addition of HRP-MBs conjugated with anti-prostate specific antigen antibody (ab10184). The solution mixture was incubated at 37 °C for 30 minutes, followed by washing the wells three times. After that, hydrogen peroxide and sodium iodide in the buffer solution (pH = 3) were added to reach a final volume of 100 μL, and kept to react at 37 °C for 1 hour.

The mixture was then taken out for the etching experiment, and the SPR absorption of gold nanostars was measured using a plate reader (SpectraMax M5, Molecular Devices).

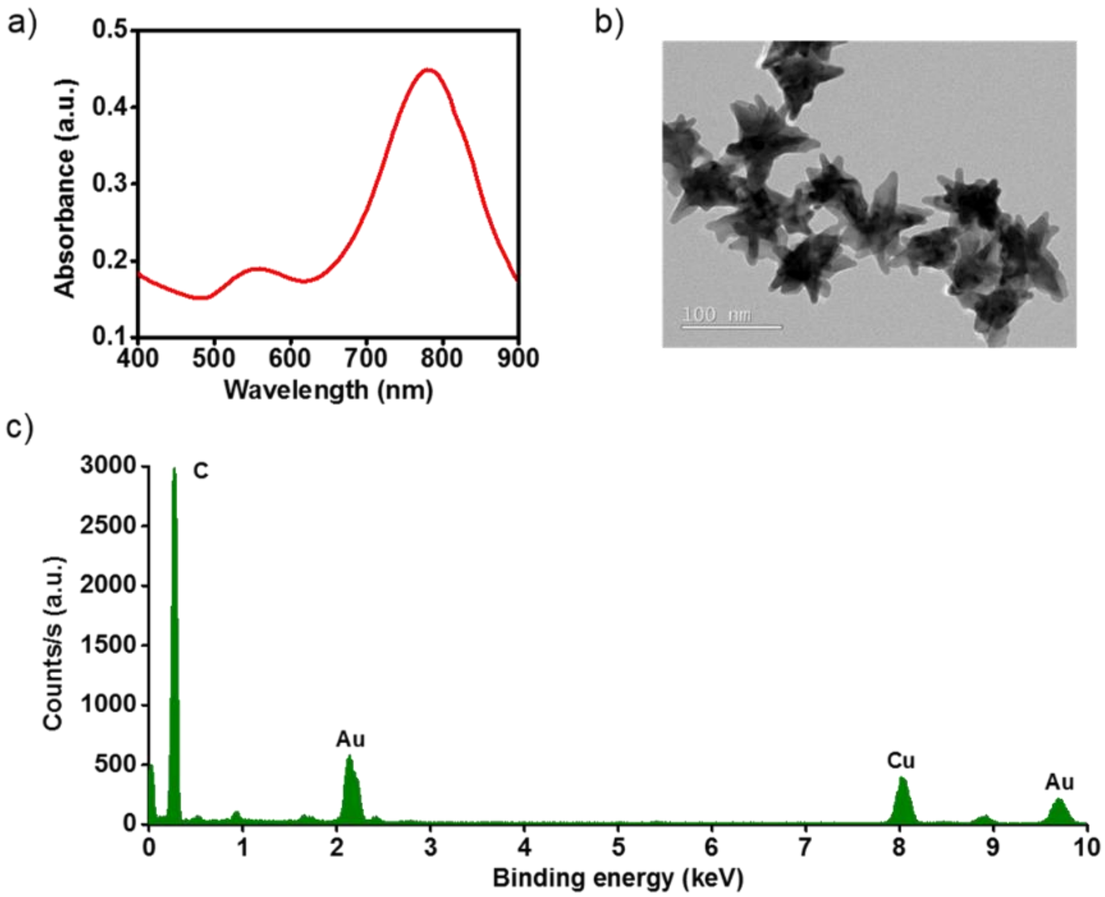

Figure S1 Characterisation of gold nanostars. (a) UV-Vis spectrum of gold nanostars. (b) TEM image of gold nanostars. (c) EDS spectrum of gold nanostars.

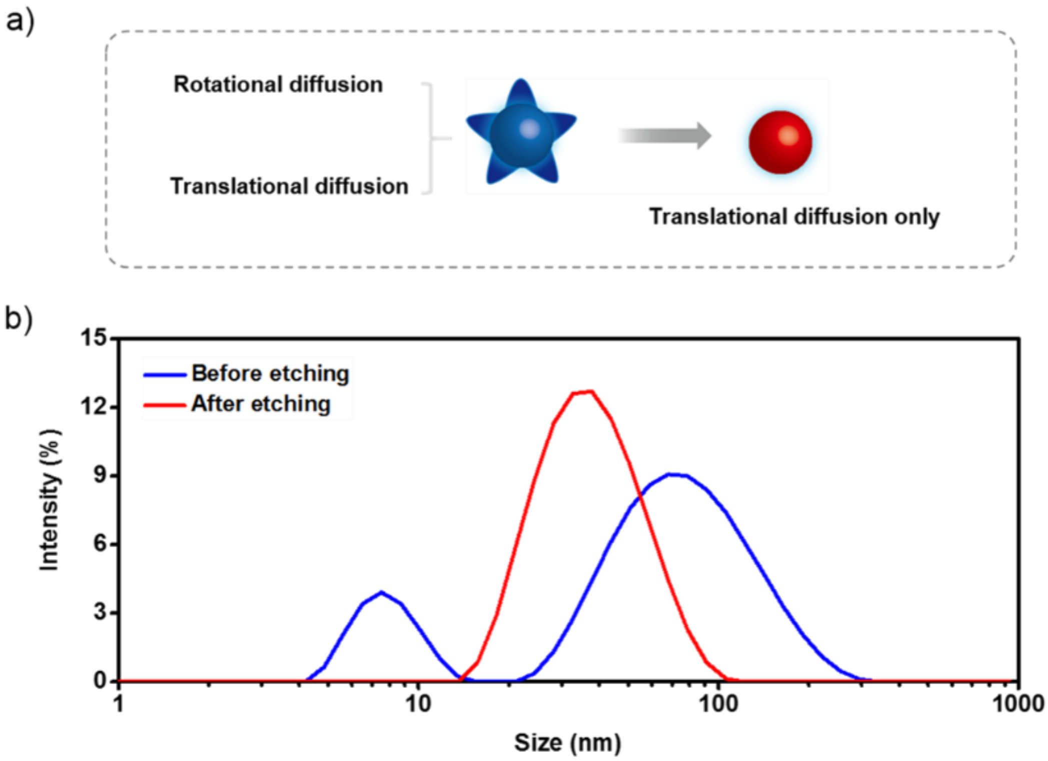

Figure S2 Dynamic light scattering characterisation of gold nanostars. (a) Schematic illustration of the different diffusion modes of star-shaped nanostructures and sphere-shaped nanostructures that results in the different dynamic light scattering signals. (b) Size distribution of gold nanostars in dynamic light scattering before and after the iodide-induced surface etching.

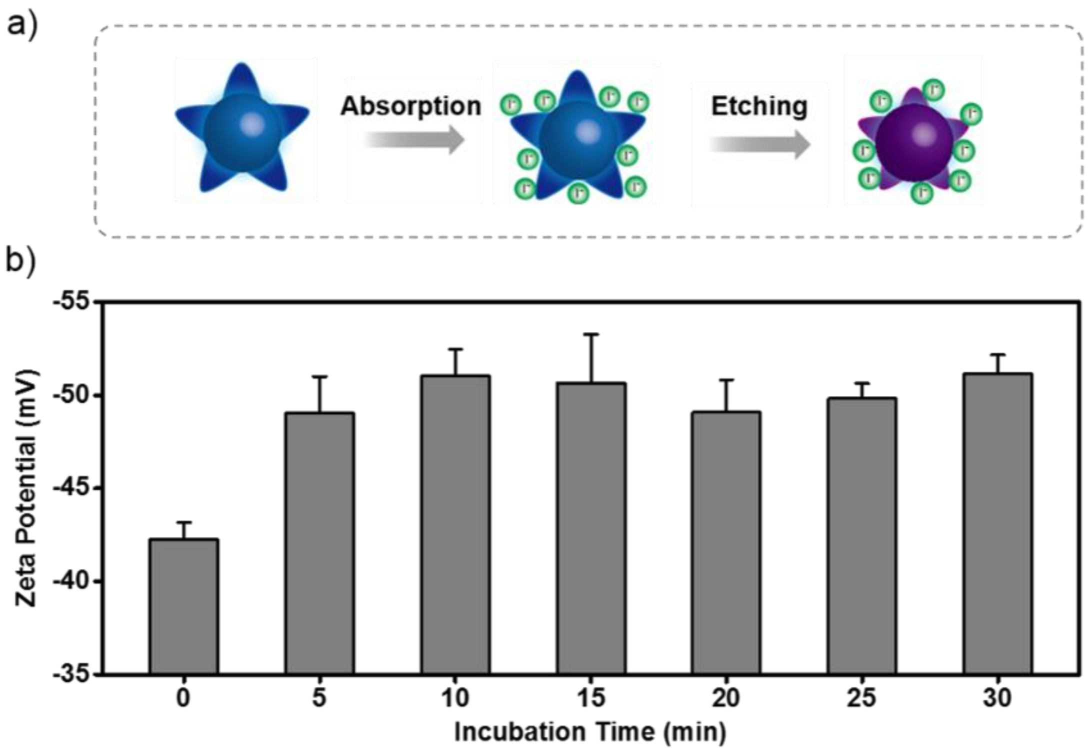

Figure S3 Iodide adsorption on the surface of gold nanostars. (a) Schematic illustration of the binding and further the etching of gold nanostars by iodide. (b) Time-dependent surface potential characterisation of gold nanostars incubated with iodide. Data represents mean ± s.d., n=3.

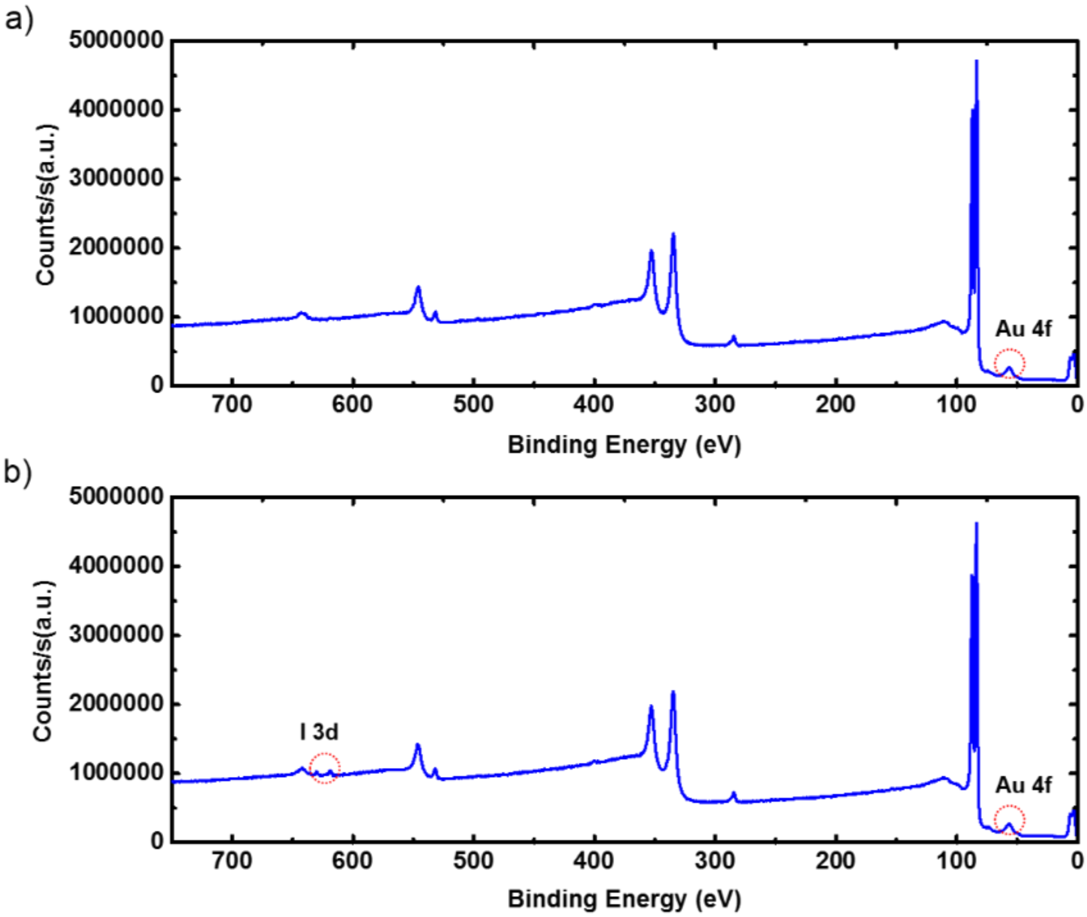

Figure S4 XPS analysis of the gold nanostars (a) before and (b) after the surface etching.

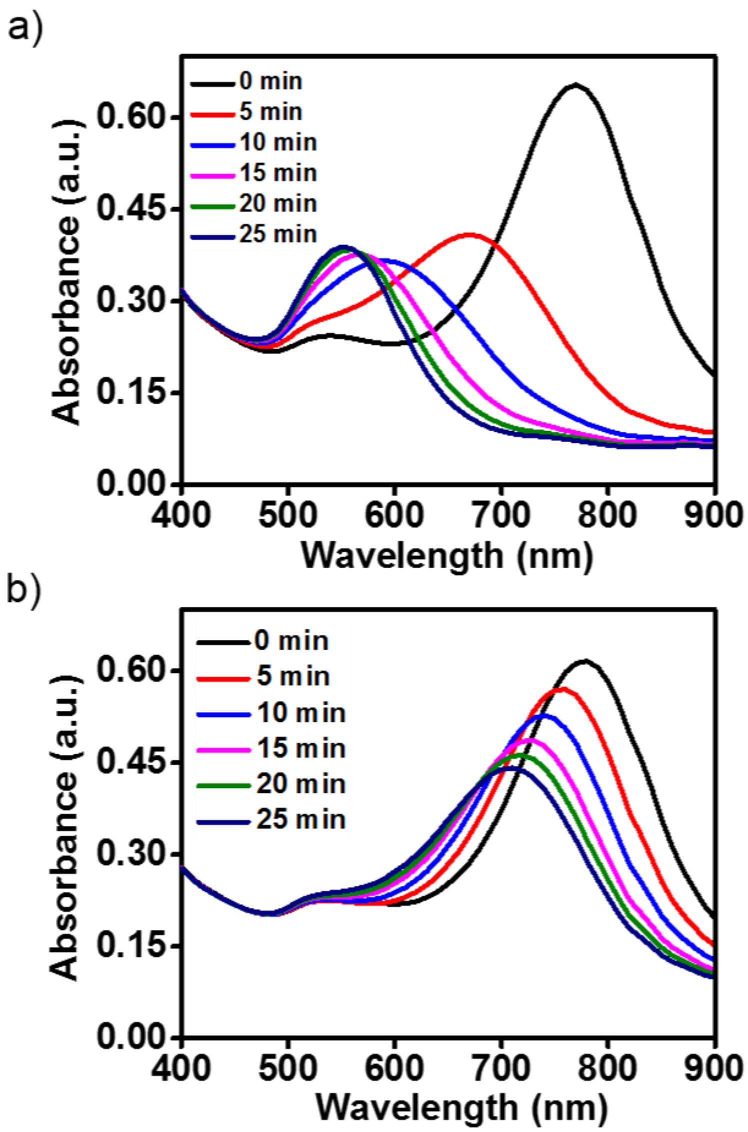

Figure S5 The effect of oxygen on the surface etching of gold nanostars. (a) Time-dependent UV-Vis spectra of gold nanostars incubated with 2 μM iodide. (b) Time-dependent UV-Vis spectra of gold nanostars incubated with 2 μM iodide in an oxygen depleted solution.

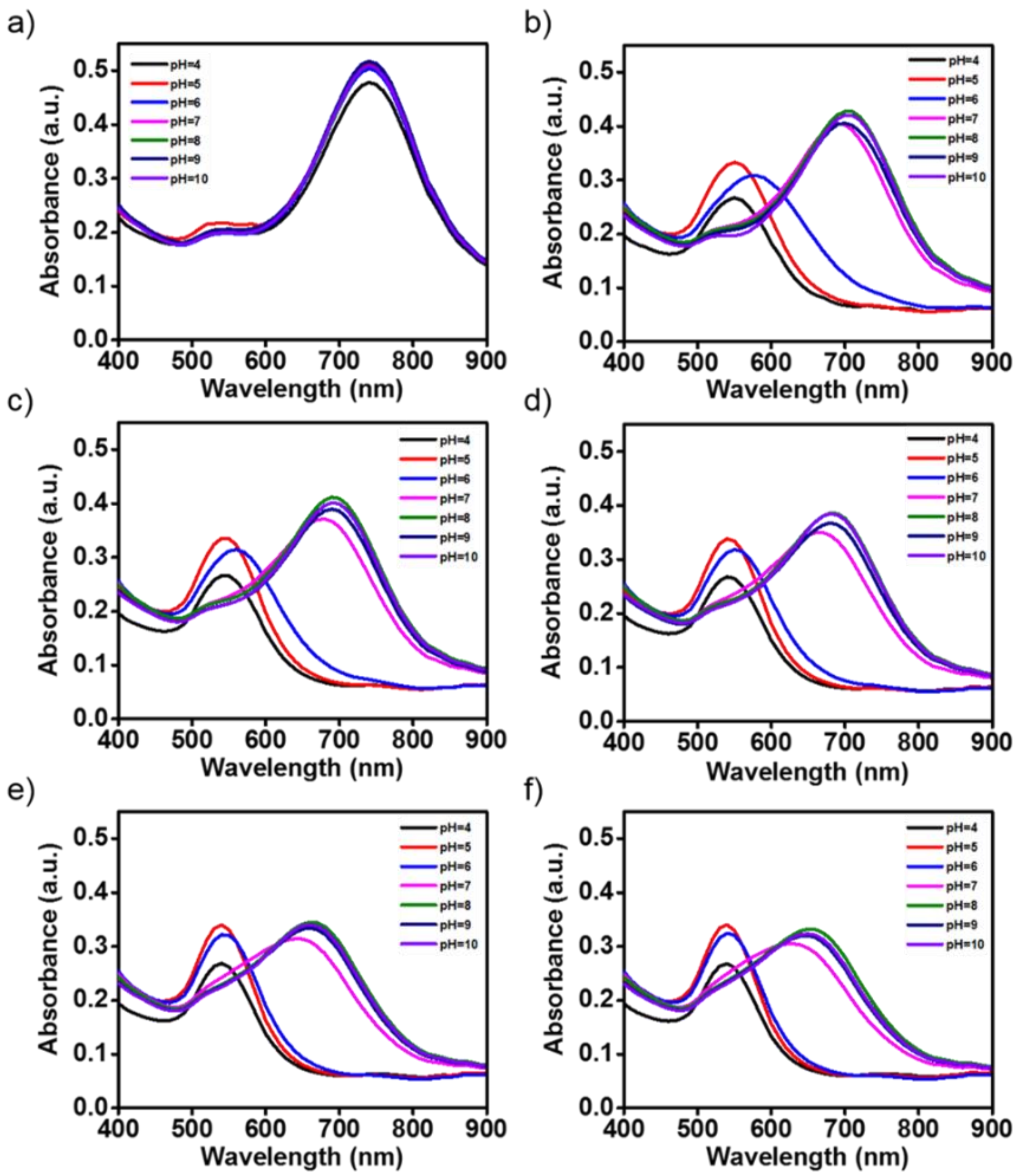

Figure S6 The effect of pH on the surface etching of gold nanostars. (a) UV-Vis spectra of gold nanostars at 0 min under different pH values (pH = 4, 5, 6, 7, 8, 9, 10). (b) to (f) Time-dependent UV-Vis spectra of gold nanostars etched by 2 μM iodide under different pH values (pH = 4, 5, 6, 7, 8, 9, 10). (b) 5 min, (c) 10 min, (d) 15 min, (e) 25 min, (f) 30 min.

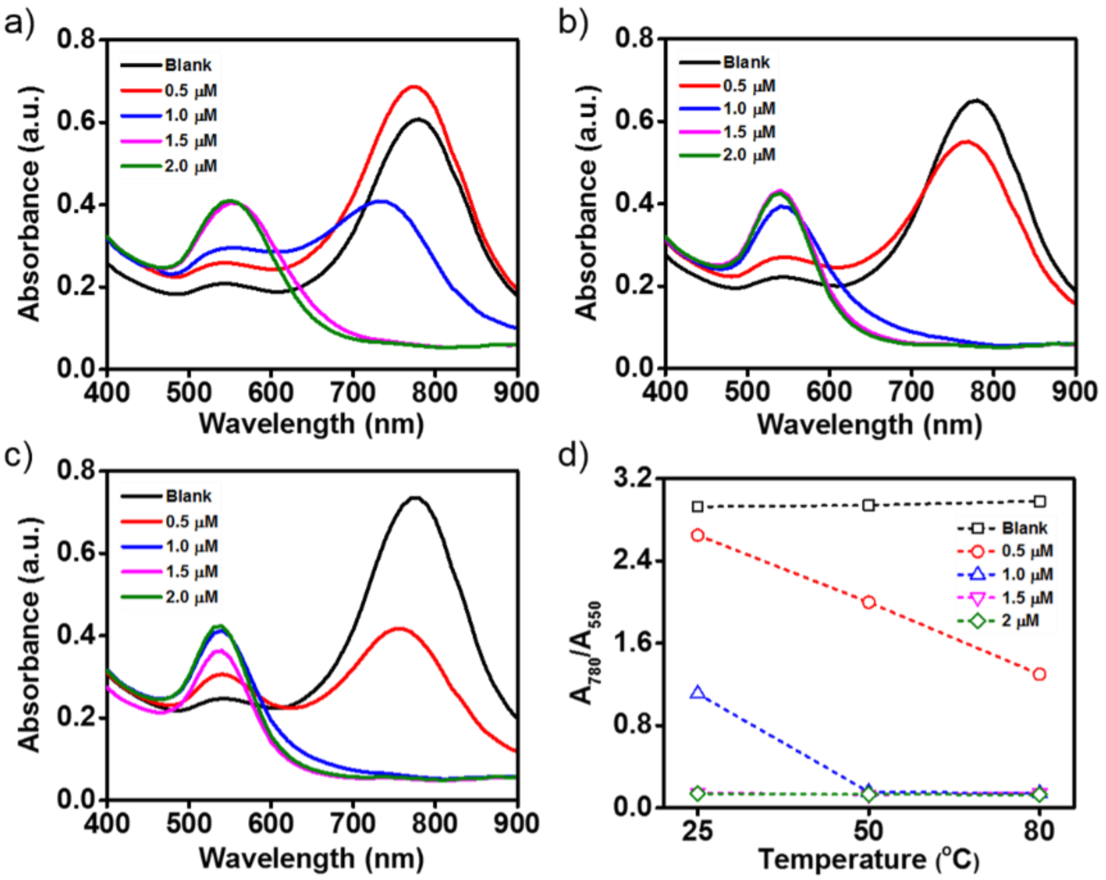

Figure S7 The effect of temperature on the surface etching of gold nanostars. UV-Vis spectra of gold nanostars etched under different temperatures with varying concentrations of iodide (0, 0.5, 1.0, 2.0 μM of iodide, etching time: 20 minutes). (a) 25 ºC, (b) 50 ºC, (c) 80 ºC, and (d) A_{780}/A_{550} values at different temperatures.

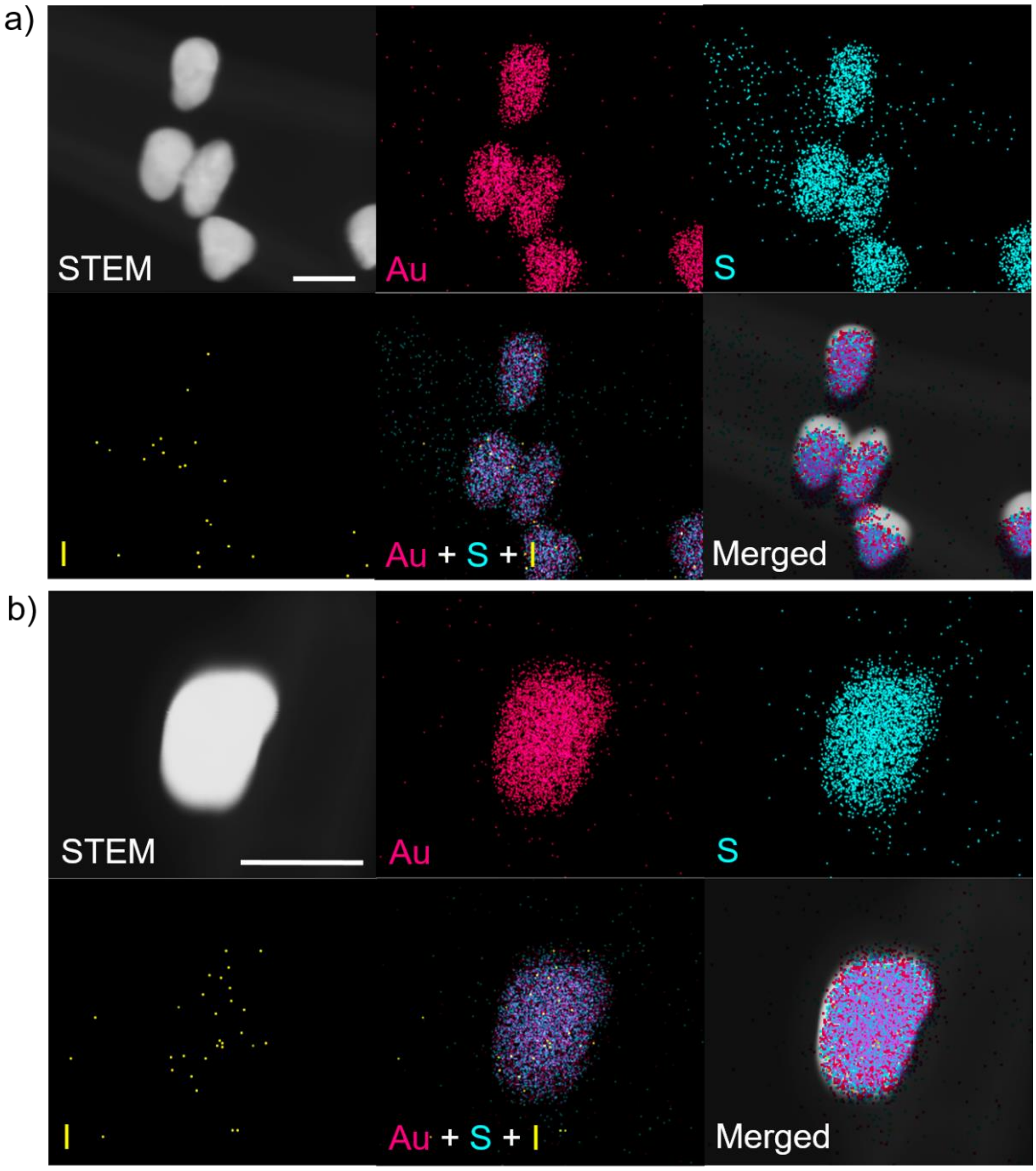

Figure S8 HAADF-STEM images and EDS elemental mapping of Au, S, I on (a) gold nanostars and (b) a single nanostar after etching by 1 μM iodide. EDS mapping reveals the morphological change of gold nanostars and the adsorption of iodide on their surface. Scale bar = 50 nm.

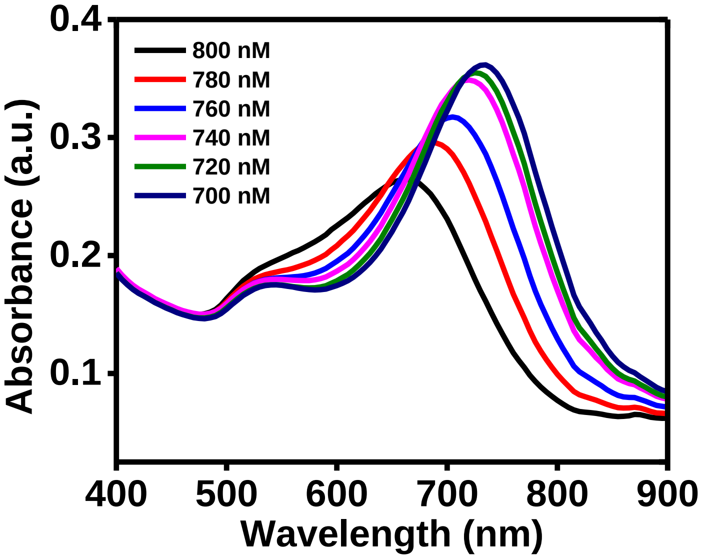

Figure S9 UV-Vis spectra of gold nanostars etched by iodide ranging from 700 nM to 800 nM.

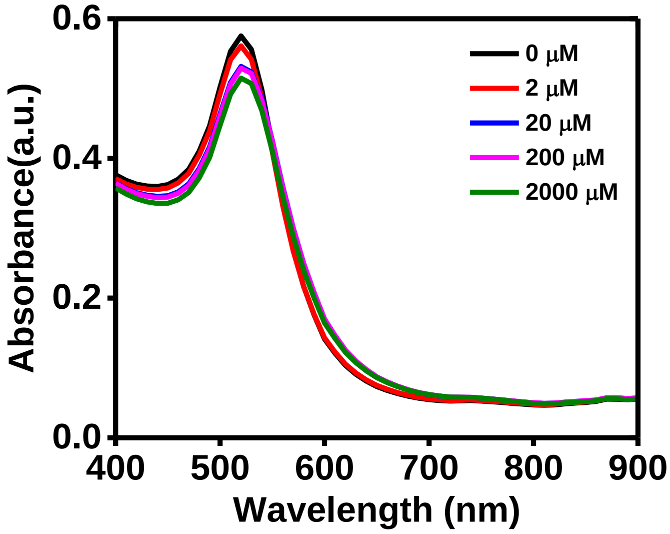

Figure S10 UV-Vis spectra of gold nanospheres etched by different concentrations of iodide ranging from 0 μM to 2000 μM. Spherical gold nanoparticles were incubated with sodium iodide for 20 minutes at room temperature.

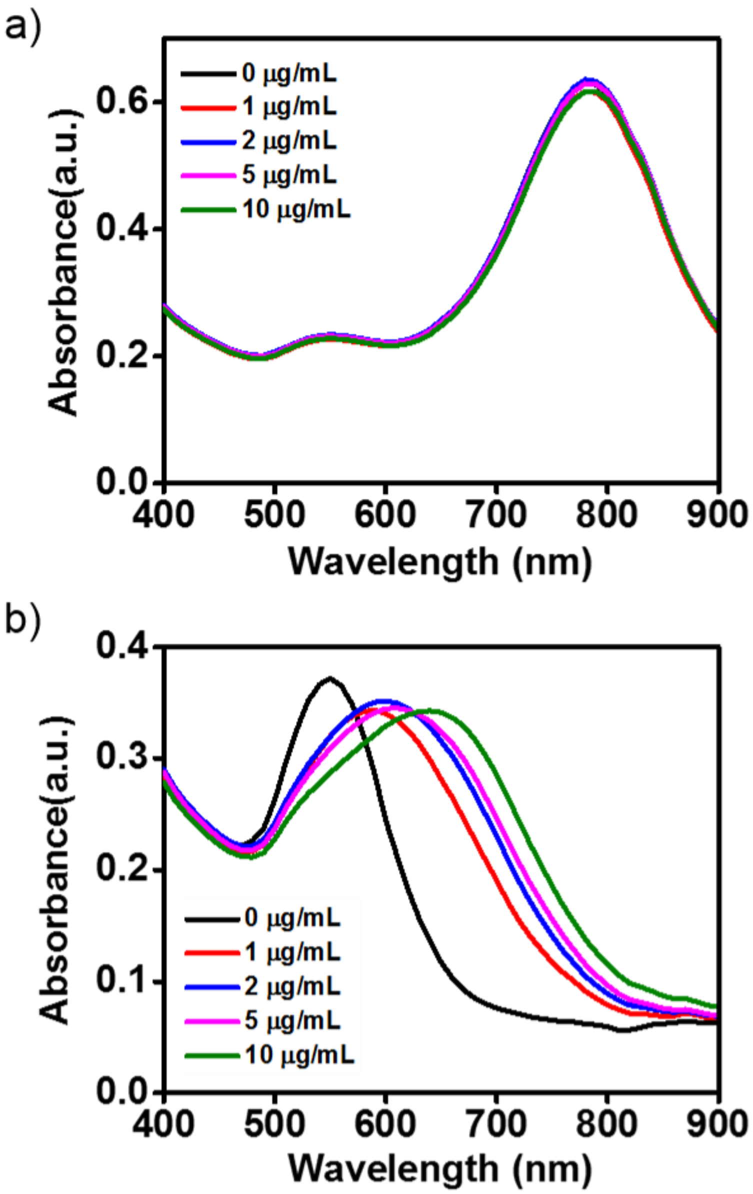

Figure S11 UV-Vis spectra of gold nanostars blocked by different concentrations of polyvinylpyrrolidone (PVP) ranging from 0 μg/mL to 10 μg/mL. (a) UV-Vis spectra of PVP-blocked gold nanostars before etching. (b) UV-Vis spectra of PVP-blocked gold nanostars after the surface etching by 2 μM iodide for 20 minutes at room temperature.

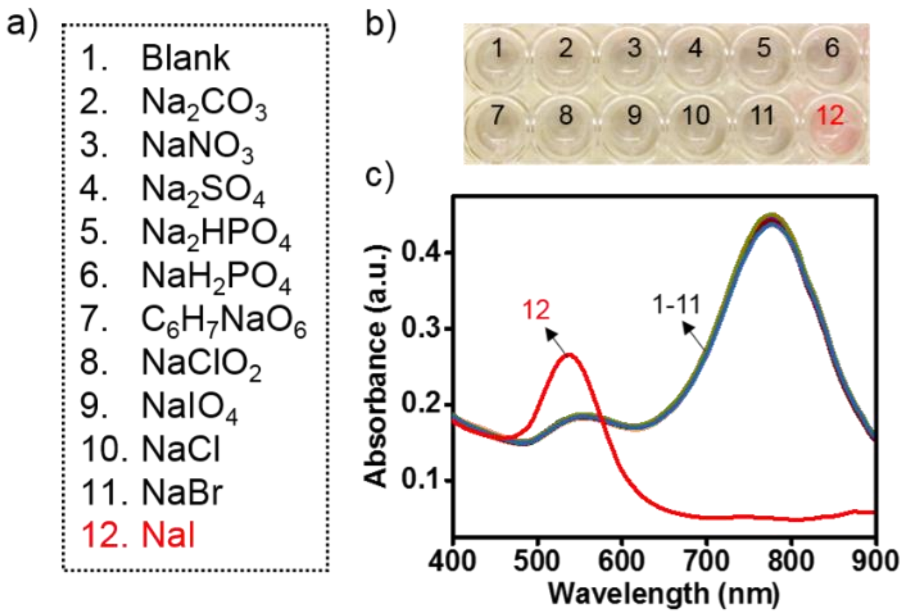

Figure S12 Surface etching of gold nanostars by iodide and other anions. (a) Sodium salts with different anions used in the etching experiment. (b) Photograph of the solution of gold nanostars incubated with sodium iodide (2 μM) and sodium salts with other anions (20 μM). (c) UV-Vis spectra of gold nanostars incubated with sodium iodide (2 μM) and sodium salts with other anions (20 μM).

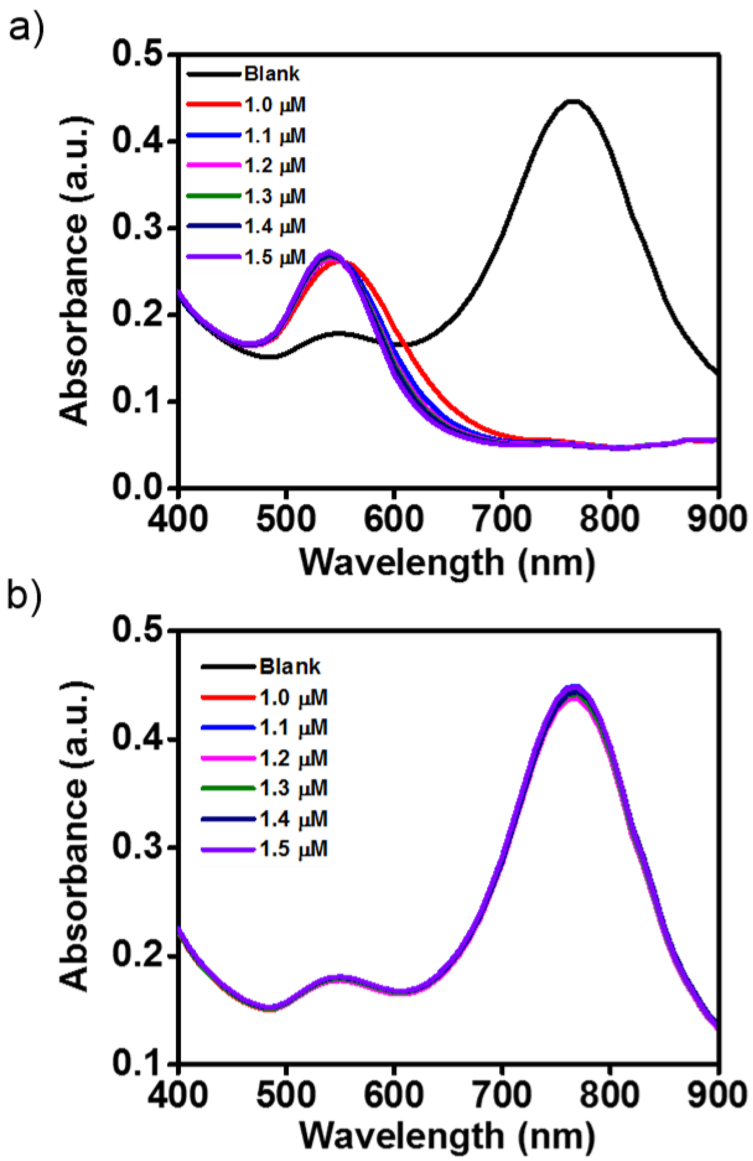

Figure S13 Effects of iodide (I^-) and iodine (I_2) on the plasmonic spectrum of gold nanostars. (a) UV-Vis spectra of gold nanostars incubated with different concentrations of iodide (0, 1.0, 1.1, 1.2, 1.3, 1.4, 1.5 µM). (b) UV-Vis spectra of gold nanostars incubated with different concentrations of iodine (0, 1.0, 1.1, 1.2, 1.3, 1.4, 1.5 µM).

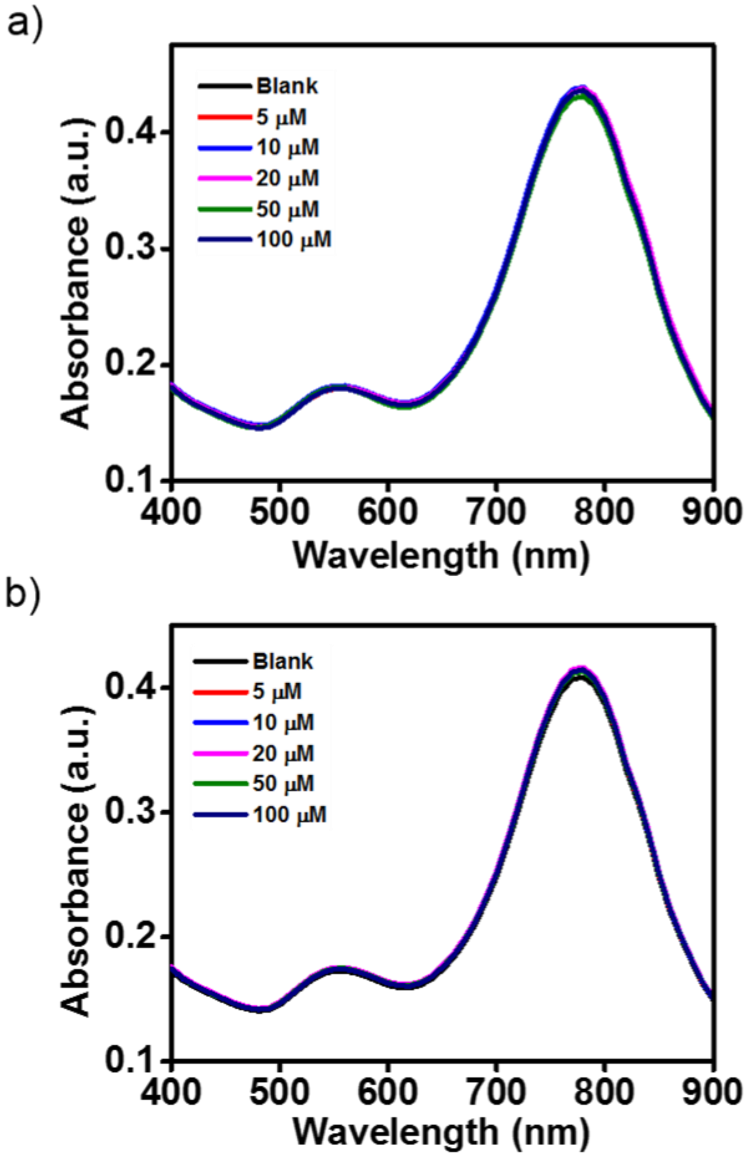

Figure S14 Effects of H_2O_2 and HCl on the plasmonic spectrum of gold nanostars. (a) UV-Vis spectra of gold nanostars incubated with different concentrations of H_2O_2 (5, 10, 20, 50, 100 μM). (b) UV-Vis spectra of gold nanostars incubated with different concentrations of HCl (5, 10, 20, 50, 100 μM).

a)

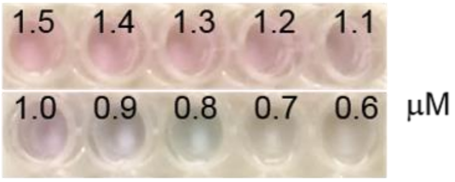

b)

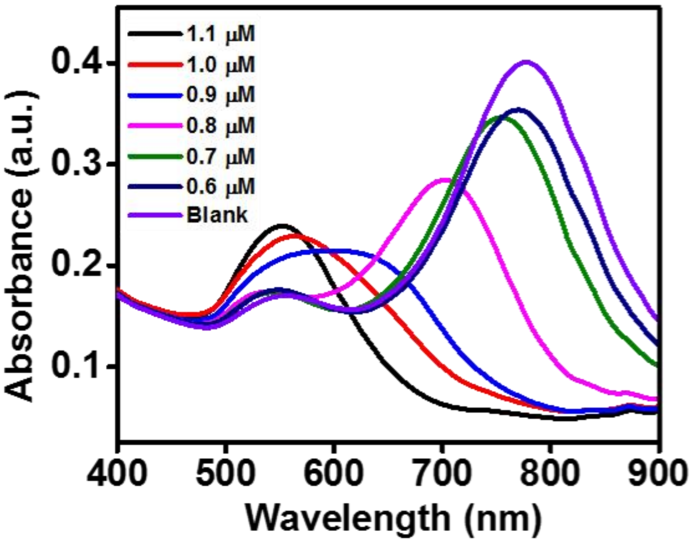

Figure S15 Surface etching of gold nanostars by iodide in the reaction buffer (pH = 3). (a) Photograph of the solution of gold nanostars etched by iodide in the reaction buffer (H_2O_2 and HCl). (b) UV-Vis spectra of gold nanostars etched by iodide ranging from 0.6 μM to 1.1 μM.

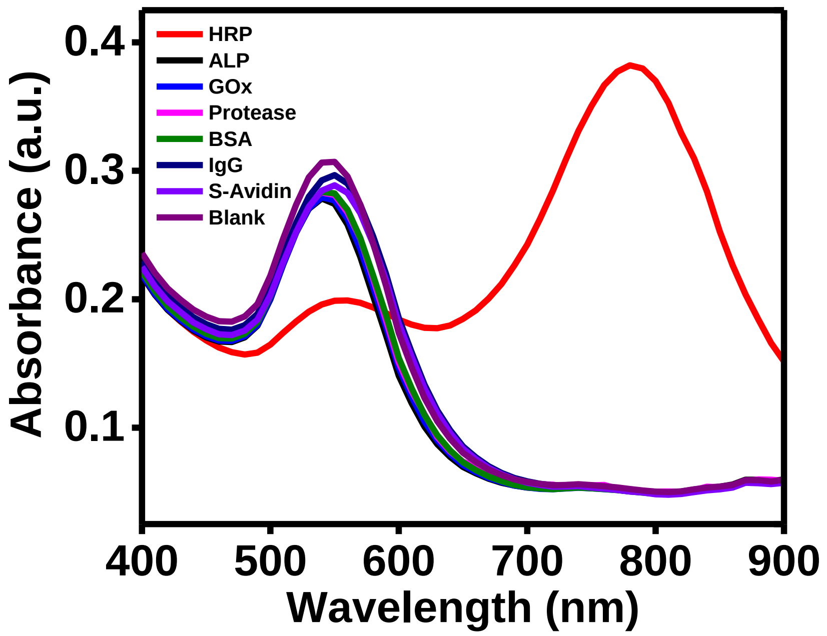

Figure S16 UV-Vis spectra of gold nanostars after the iodide consumption by the catalysis of 40 ng/mL of HRP or 800 ng/mL of other proteins: ALP, GOx, protease, BSA, IgG and streptavidin (S-Avidin).

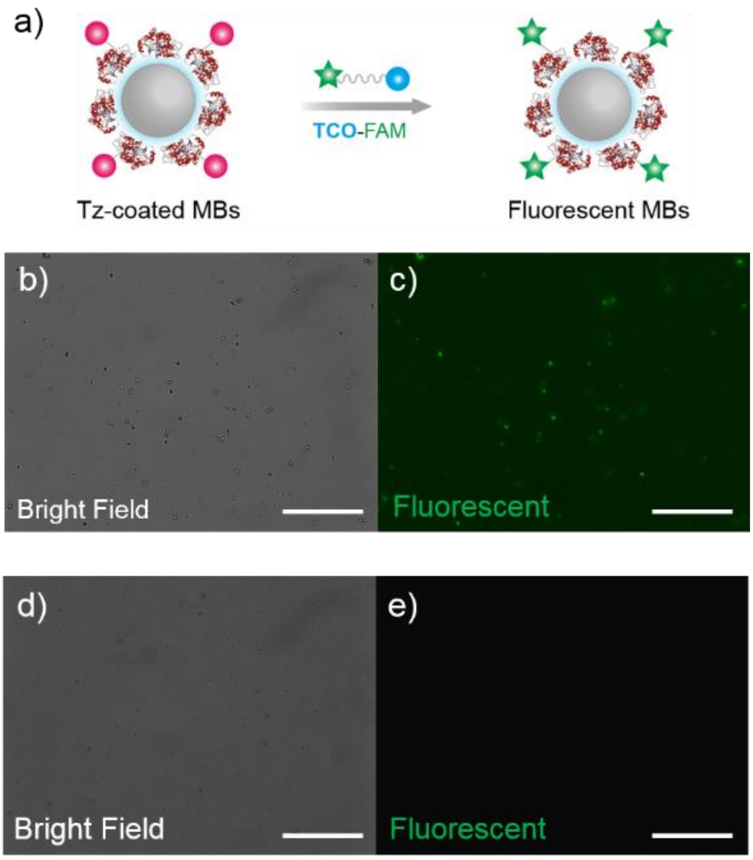

Figure S17 Fluorescent images of magnetic beads (1 μm) after incubation with TCO-functionalised fluorescent dye (TCO-FAM). (a) Schematic illustration of the bioorthogonal reaction between Tz-coated magnetic beads and TCO-FAM. (b) and (c) Bright field image and fluorescent image of the Tz-coated magnetic beads after incubation with TCO-FAM. (d) and (e) Bright field image and fluorescent image of the unmodified magnetic beads after incubation with TCO-FAM. Scale bar = 50 μm.

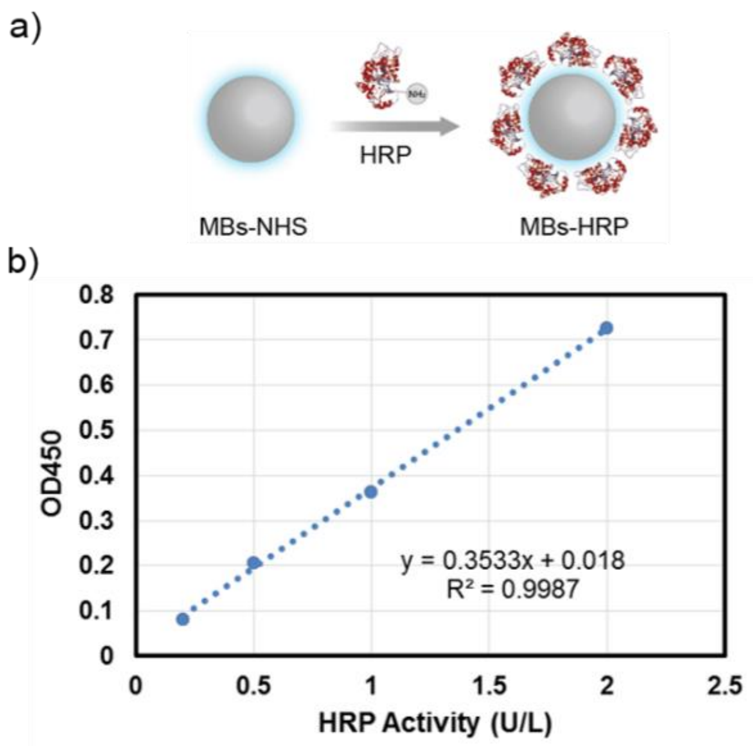

Figure S18 HRP conjugation on the NHS-activated MBs. (a) Schematic illustration of HRP modification on the magnetic beads using NHS chemistry. (b) The quantification curve for HRP based on the linear relationship between the concentration of HRP and the OD450 value using TMB as the substrate. The amount of HRP loaded on the magnetic beads can be calculated based on the quantification curve.

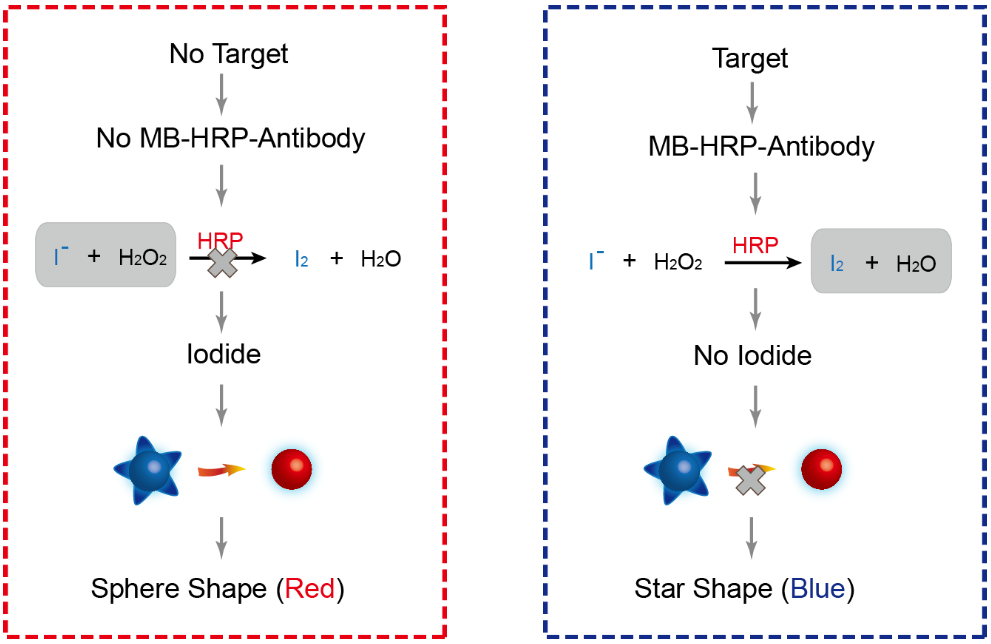

Scheme S1 Schematic illustration of the events occurred in the plasmonic immunoassay using etching-based approach.

Table S1. Comparison of nanostar etching-based method and other methods for PSA detection.

Material	Strategy	Readout	Sensitivity	Reference
Au nanorods	Surface etching	Colour change	75 pg/mL	[1]
Pd-Ir nanoparticles	Enzyme mimic	Colour intensity	31 fg/mL	[2]
Au@Pt nanoparticles	Enzyme mimic	Colour intensity	20 pg/mL	[3]
Au nanoparticles	Nano-carrier	Fluorescence	32 fg/mL	[4]
DNA tetrahedron	Microarray	Fluorescence	40 pg/mL	[5]
Magnetic nanoparticles	Surface-enhanced Raman scattering (SERS)	Raman shift	12 pg/mL	[6]
Magnetic and Au nanoparticles	Bio-barcode and nano-carrier	Grayscale	330 fg/mL	[7]
Au nanostars	Surface etching	Colour change	10 pg/mL	This study

References

[1] X. M. Ma, Y. Lin, L. H. Guo, B. Qiu, G. N. Chen, H. H. Yang, Z. Y. Lin, *Biosens Bioelectron* **2017**, *87*, 122-128.

[2] H. H. Ye, K. K. Yang, J. Tao, Y. J. Liu, Q. Zhang, S. Habibi, Z. H. Nie, X. H. Xia, *ACS Nano* **2017**, *11*, 2052-2059.

[3] Z. Q. Gao, H. H. Ye, D. Y. Tang, J. Tao, S. Habibi, A. Minerick, D. P. Tang, X. H. Xia, *Nano Lett* **2017**, *17*, 5572-5579.

[4] D. B. Liu, X. L. Huang, Z. T. Wang, A. Jin, X. L. Sun, L. Zhu, F. Wang, Y. Ma, G. Niu, A. R. H. Walker, X. Y. Chen, *ACS Nano* **2013**, *7*, 5568-5576.

[5] Z. H. Li, B. Zhao, D. F. Wang, Y. L. Wen, G. Liu, H. Q. Dong, S. P. Song, C. H. Fan, *ACS Appl Mater Inter* **2014**, *6*, 17944-17953.

[6] Z. Cheng, N. Choi, R. Wang, S. Lee, K. C. Moon, S. Y. Yoon, L. X. Chen, J. Choo, *ACS Nano* **2017**, *11*, 4926-4933.

[7] C. S. Thaxton, R. Elghanian, A. D. Thomas, S. I. Stoeva, J. S. Lee, N. D. Smith, A. J. Schaeffer, H. Klocker, W. Horninger, G. Bartsch, C. A. Mirkin, *Proc. Natl. Acad. Sci. U.S.A.* **2009**, *106*, 18437-18442.
